# Functional aortic stiffness: role of CD4^+^ T lymphocytes

**DOI:** 10.3389/fphys.2015.00235

**Published:** 2015-08-26

**Authors:** Beenish A. Majeed, Lance S. Eberson, Supannikar Tawinwung, Nicolas Larmonier, Timothy W. Secomb, Douglas F. Larson

**Affiliations:** ^1^Sarver Heart Center, College of Medicine, The University of ArizonaTucson, AZ, USA; ^2^Department of Pharmacology, College of Medicine, The University of ArizonaTucson, AZ, USA; ^3^Departments of Pediatrics and Immunology, College of Medicine, The University of ArizonaTucson, AZ, USA; ^4^Department of Physiology, College of Medicine, The University of ArizonaTucson, AZ, USA; ^5^Department of Surgery, College of Medicine, The University of ArizonaTucson, AZ, USA

**Keywords:** pulse wave velocity, angiotensin II, vascular remodeling, vasoconstriction, lymphocytes

## Abstract

The immune system is suggested to be essential in vascular remodeling and stiffening. To study the dependence upon lymphocytes in vascular stiffening, we compared an angiotensin II-model of vascular stiffening in normal C57BL/6J mice with lymphocyte-deficient RAG 1^−/−^ mice and additionally characterized the component of vascular stiffness due to vasoconstriction vs. vascular remodeling. Chronic angiotensin II increased aortic pulse wave velocity, effective wall stiffness, and effective Young's modulus in C57BL/6J mice by three-fold but caused no change in the RAG 1^−/−^ mice. These functional measurements were supported by aortic morphometric analysis. Adoptive transfer of CD4^+^ T helper lymphocytes restored the angiotensin II-mediated aortic stiffening in the RAG 1^−/−^ mice. In order to account for the hydraulic vs. material effects of angiotensin II on pulse wave velocity, subcutaneous osmotic pumps were removed after 21 days of angiotensin II-infusion in the WT mice to achieve normotensive values. The pulse wave velocity (PWV) decreased from three- to two-fold above baseline values up to 7 days following pump removal. This study supports the pivotal role of the CD4^+^ T-lymphocytes in angiotensin II-mediated vascular stiffening and that angiotensin II-mediated aortic stiffening is due to the additive effect of active vascular smooth muscle vasoconstriction and vascular remodeling.

## Introduction

Progressive aortic stiffening predisposes individuals to cardiac, cerebral, and renal dysfunction (Boutouyrie et al., 2002; Han et al., 2013; Tsao et al., 2013; Yoon et al., 2013). A mechanism of injury suggests that arterial stiffening increases pulsatile flow and pressure patterns, thereby injuring the microvasculature of highly perfused organs, particularly the brain and kidneys. In addition, stiffening causes the incident and reflected flow and pressure waves to travel faster, leading to early return of the reflected waves that functionally cause increased left-ventricular afterload and eventually diastolic dysfunction. Due to the wide array of diseases attributed to this vascular condition, there is a recognized clinical need to investigate the pathogenesis of arterial vascular stiffening and to provide research lines that will reduce the disease burden caused by vascular stiffness.

Functional or effective arterial stiffness is determined in the human primarily with pulse wave velocity (PWV) and in the rodent models with *ex vivo* stress/strain determination. The *in vivo* PWV measurements blend the effects of vascular pressure, vascular smooth muscle (VSMC) tone, pre-strain, and diameter with the material composition of the extracellular matrix (ECM) in the determination of vascular stiffness. Conversely, *ex vivo* measurements using sequential biaxial loading includes the passive biomechanical properties that relate to ECM material composition. It has been recently noted that the VSMCs play an active role in and are integral regulators of aortic stiffness which is generally not characterized in the experimental models using *ex vivo* biomechanical analyses (Saphirstein and Morgan, 2014). Thus, both accepted methods of assessing vascular stiffness using *in vivo* PWV and *ex vivo* biaxial biomechanics provide descriptive data regarding vascular stiffness but are limited in their ability to differentiate VSMC contribution separately from the ECM biomaterial composition to vascular stiffness.

VSMC responses to angiotensin II (Ang II) infusion appear to be independent of adaptive immunity (Majeed et al., 2014). However, the ECM material composition and function is dependent upon the competence of the adaptive immune system. Furthermore, the adaptive immune system has been shown to be an important component of Ang II-related vascular disease. RAG 1^−/−^ mice, lacking T- and B-lymphocytes, have been shown to exhibit blunted responses to Ang II in hypertension and vascular stiffness (Guzik et al., 2007; Wu et al., 2014). However, there is a requirement to integrate VSMC responses with ECM remodeling responses to appreciate the vascular mechanics and physiology.

T cells have been shown to increase vascular remodeling in RAG 1^−/−^ mice and stiffness as measured by *ex vivo* stress–strain relationships (Wu et al., 2014). We sought to confirm these findings in a clinically relevant method of measuring stiffness: PWV. We also investigated the diastolic flow fraction and reflective wave propagation of the aortic flow Doppler to further characterize the effects of stiffening on arterial function. In this study, we assess the effect of changes in blood pressure and ECM remodeling by collagen deposition as they contribute to *in vivo* measurements of vascular stiffness. Moreover, we show specifically that CD4^+^ T cells support the vascular stiffening process.

## Methods

### Animals

C57BL/6J, RAG 1^−/−^, and CD8^−/−^ male mice (8-week old) were obtained from the Jackson Laboratories (Bar Harbor, ME, USA). All of mice used in this study were of C57BL/6J background. This study was approved by the University of Arizona Animal Care Committee and conforms to the Guide for the Care and Use of Laboratory Animals published by the US National Institutes of Health (NIH Publication No. 85-23, revised 1996). All the mice were maintained in the animal facility of the University of Arizona and fed with NIH-31 Modified Open Formula Mouse/Rat Sterilizable Diet from Harlan Laboratories. The mice were treated with [Val^5^]angiotensin II (Ang II) (Sigma-Aldrich) at 490 ng/min/kg via subcutaneously implantable osmotic pumps (model 1004; Alzet, Palo Alto, CA, USA). The mice were then monitored for up to 21 days of Ang II infusion until sacrifice.

### Tail cuff

All mice were trained with the tail cuff system (Hatteras Instruments, Cary, North Carolina) and data were recorded for days 0, 7, 14, and 21. Tail cuff measurements were performed with the mice on a heated platform. Blood pressure values were recorded from an average of 10 consecutive measurements with standard deviation lower than 10.

### ECHO/doppler

PWV, reflective wave, and diastolic flow fraction were calculated from noninvasive ECHO Doppler measurements acquired with the Vevo 770 High Resolution Imaging System (VisualSonics, Toronto, Canada). Estimation of wall stiffness, Eh, Young's modulus, E, and wall thickness, h, were derived from the Moens–Korteweg equation for PWV. Detailed methods are described previously (Eberson et al., 2015).

### Diastolic flow fraction and reflective wave

Diastolic flow fraction was calculated as the percentage of the velocity-time integral of the Doppler trace of the aortic flow wave in diastole superior to the renal bifurcation. Reflected flow waves have the same shape as the pressure waves but are 180° out of phase (Westerhof et al., 1972). We measured the distance from the EKG R-wave to the initial beginning of the innominate lower extremity flow wave (Heffernan et al., 2013) and presented it as the transit time of the reflective wave (ms).

### Perfusion fixation and histological staining

Before perfusion fixation, mice were injected with 100 μl of 1000 USP units/ml Heparin sodium. Thirty milliliters of saline was infused to remove vascular blood, followed by the administration of 2:1 3% glutaraldehyde:1% formaldehyde solution at a constant aortic perfusion pressure of 40 mmHg for 15 min. This technique reduces fixation contraction. Aortic tissues were paraffin embedded, cut at 5 μm, and stained with Masson's Trichrome and Picrosirius red for morphology and collagen assessment with NIH ImageJ. The area of Picrosirius red staining was normalized to the lumen diameter.

### T cell isolation and adoptive transfer

Enriched T cells were isolated from spleens and lymph nodes of CD8^−/−^ C57BL/6 mice (red blood cell lysis followed with IgG panning). Flow cytometry was used to confirm 98% purity of CD4^+^ T lymphocytes. 2 × 10^7^ isolated CD4^+^ T cells were injected intra-peritoneally into each RAG 1^−/−^ mouse 2 weeks prior to Ang II administration.

### Flow cytometry

Isolated lymphocytes were stained with cell surface antibodies. Efluor 450-conjugated anti-CD3, APC-conjugated anti-CD19, Efluor450-conjugated anti-CD4, and FITC-conjugated anti-CD8a were purchased from eBioscience. The BD LSR II with BD FACSDiva software was used to analyze data.

### Statistics

All data are reported as means ± SEM. Comparisons among the defined groups were analyzed by One-Way ANOVA, followed by Tukey multiple comparisons tests using GraphPad Prism. *P* < 0.05 was considered statistically significant.

## Results

### Aortic stiffness is reduced in RAG 1^−/−^ mice

**Figure 1:**
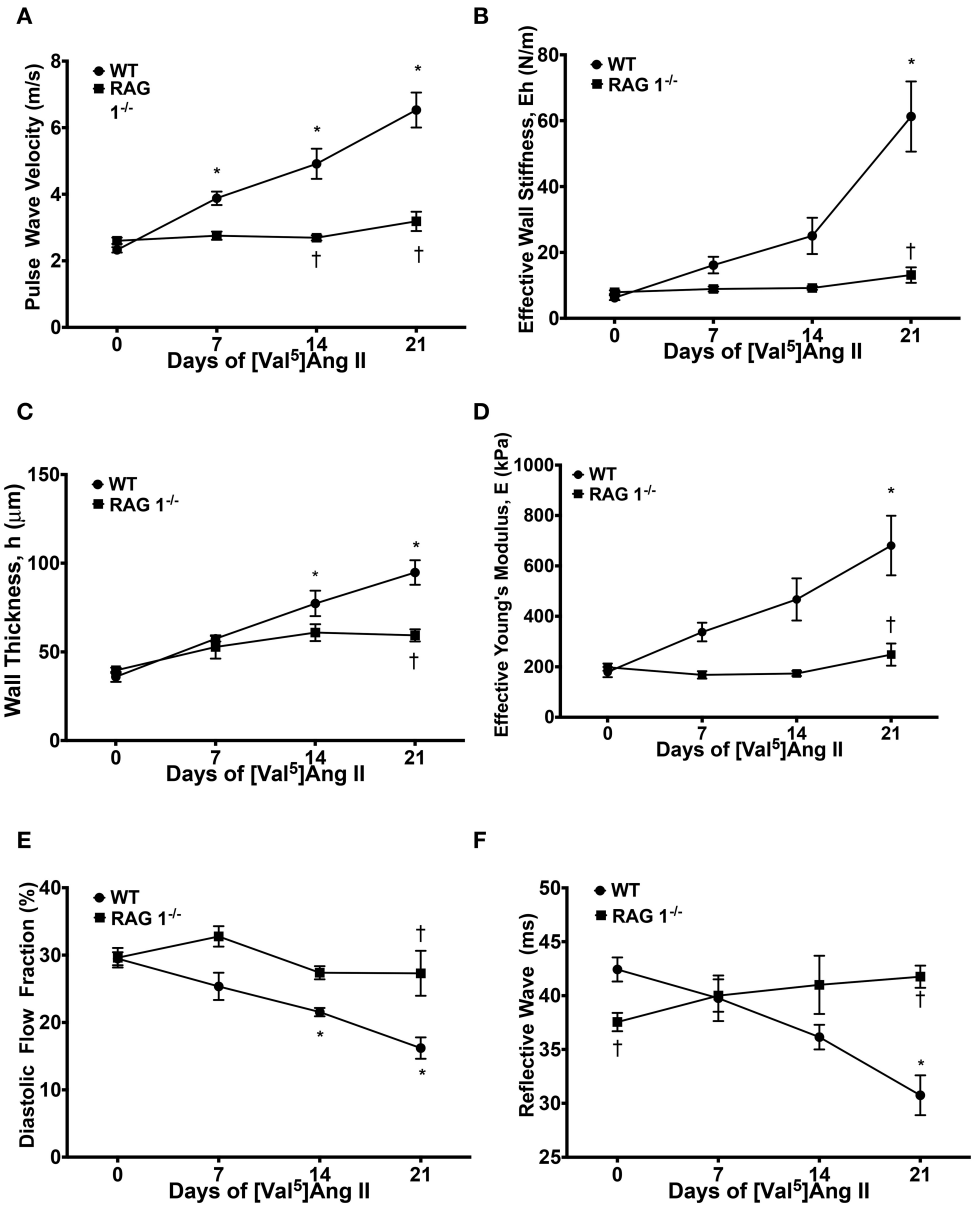
**Stiffness measurements**. Mice were treated with [Val^5^]Ang II at 490 ng/kg/min for 3 weeks. **(A)** Pulse wave velocity (PWV) in response to Ang II treatment between WT and RAG 1^−/−^ mice was measured. PWV was used to calculate **(B)** effective wall stiffness, **(C)** wall thickness, and **(D)** effective Young's modulus, E. **(E)** Diastolic flow fraction was calculated as the diastolic percentage of the velocity-time-integral (VTI) in the aortic Doppler flow wave. **(F)** Reflective wave represents the transit time of the intersection of the forward and reflective flow waves from the R wave of the EKG. ^*^P < 0.05 to day 0 WT; ^†^*P* < 0.05 to WT at respective time point. *N* = 4–22. Data are means ± SEM.

We first determined if immunodeficient RAG 1^−/−^ mice lacking mature T- and B-lymphocytes would demonstrate aortic vascular stiffness comparable with that of strain-matched immunocompetent mice. Central aortic stiffness was measured by PWV in WT and RAG 1^−/−^ mice. Baseline PWV-values were similar in WT and RAG 1^−/−^ mice (2.3 ± 0.4 vs. 2.6 ± 0.3 m/s). With chronic Ang II infusion, PWV increased in WT mice to 6.5 ± 0.6 m/s by day 21 but no increase in PWV was detected in the RAG 1^−/−^mice (Figure 1A). Similarly the calculated effective wall stiffness (Eh) increased in the WT but not in the RAG 1^−/−^ mice (Figure 1B). Aortic wall thickness (h) increased in both strains but this increase was significantly reduced in the RAG 1^−/−^ mice (Figure 1C). Finally, effective Young's modulus (E) increased over the 21-day treatment of Ang II in the WT but not in the RAG 1^−/−^ mice (Figure 1D). Additional parameters of stiffness using aortic flow Doppler were measured. WT mice exhibited a significant decrease in diastolic flow fraction by day 21, while RAG 1^−/−^ showed no significant change (Figure 1E). This decrease in diastolic flow percentage of the Doppler trace is related to the loss in Windkessel function of the aorta with chronic Ang II treatment. Additionally, the transit time of the reflective wave was reduced by day 21 in WT mice but not in RAG 1^−/−^ mice (Figure 1F). This is supported by a change in flow wave reflection with vascular stiffness (Murgo et al., 1981). The unexpected difference in reflective wave at day 0 could possibly be due to developmental differences between the strains.

The increase in the effective Young's modulus in the WT group is dependent upon the material composition and/or mechanical properties of the wall rather than the changes in vascular morphometric properties. Furthermore, the Ang II-mediated increase in Young's modulus in the WT but not in the RAG 1^−/−^ mice in response to Ang II implies its dependency upon immune cells.

### Blood pressure in WT and RAG 1^−/−^ mice

**Figure 2:**
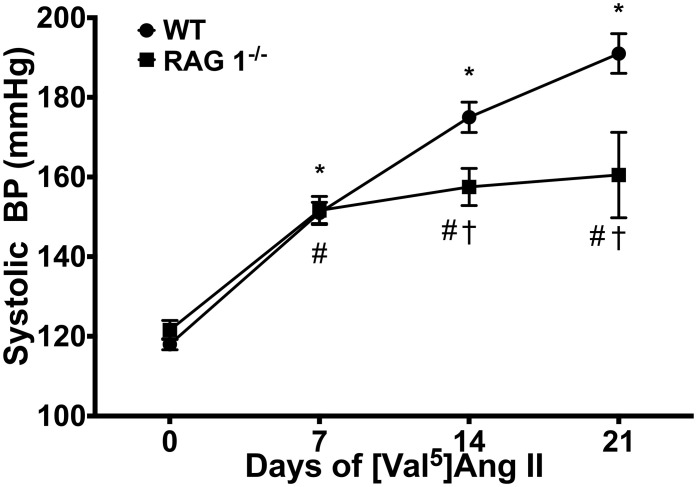
**Blood pressure**. Systolic blood pressures were measured by tail cuff in WT and RAG 1^−/−^ over the [Val^5^]Ang II treatment. *N* = 15–47. ^*^P < 0.05 vs. day 0 of WT; ^#^*P* < 0.05 vs. day 0 of RAG 1^−/−^; ^†^*P* < 0.05 between different strains at each time point. Data are means ± SEM.

Blood pressure was measured in mice receiving chronic Ang II infusion. WT and RAG 1^−/−^ mice exhibited a significant increase in systolic blood pressure as measured by tail cuff over 21 days (Figure 2). By 14 days, RAG 1^−/−^ mice had an average systolic pressure that was significantly lower than that of WT (158 ± 4.7 vs. 175 ± 3.8 mmHg). The difference in pressures remained significant at 21 days (161 ± 10.7 vs. 191 ± 5.0 mmHg).

### The ang II-induced increase in *in vivo* measures of stiffness is due to vascular remodeling

**Figure 3:**
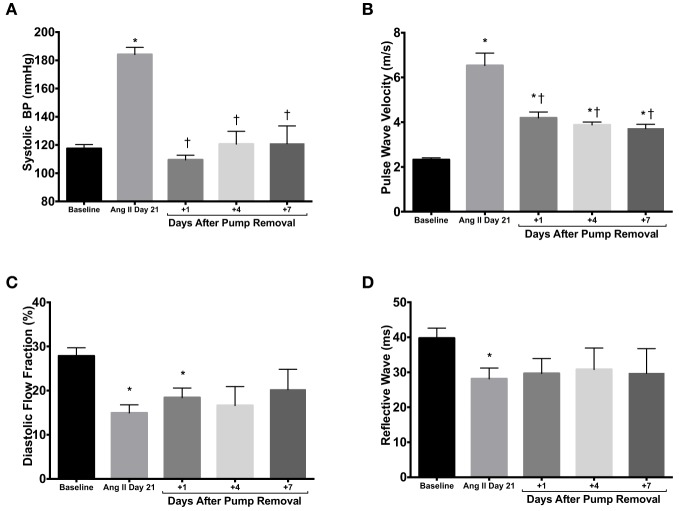
**Pump removal. (A)** After 21 days of Ang II treatment, the osmotic pumps releasing Ang II were removed and systolic blood pressure was measured 1, 4, and 7 days thereafter. Stiffness parameters of PWV **(B)**, diastolic flow fraction **(C)**, and reflective wave **(D)** were also measured. ^*^P < 0.05 vs. day 0; ^†^*P* < 0.05 vs. day 21. *N* = 4–6. Data are means ± SEM.

In order to account for the differential effects of Ang II on PWV, namely vasoconstriction as opposed to vascular remodeling, we removed the subcutaneous osmotic pumps after 21 days of Ang II infusion in the WT mice. Systolic blood pressure dropped immediately to normotensive values 1 day after pump removal (Figure 3A). Following the three-fold increase in PWV values with 21 days of Ang II administration, PWV remained two-fold above baseline values at 1, 4, and 7 days after pump removal (Figure 3B). Pumps were also removed after treatment in RAG 1^−/−^ but no changes were observed in PWV (data not shown). Diastolic flow fraction remained significantly lower than baseline values 1 day after pump removal (Figure 3C), although reflective wave showed no significant differences after Ang II stimulus-removal compared to baseline levels (Figure 3D).

Taken together, these data reveal that under the conditions described above the functional measurement of vascular stiffness is due to both the vasoconstrictive effect of Ang II and Ang II-mediated vascular remodeling. These pump removal data are consistent with a recent report supporting the significant role of vascular smooth muscle in vascular stiffening (Saphirstein and Morgan, 2014).

### Ang II-induced vascular remodeling is reduced in RAG 1^−/−^ mice

**Figure 4:**
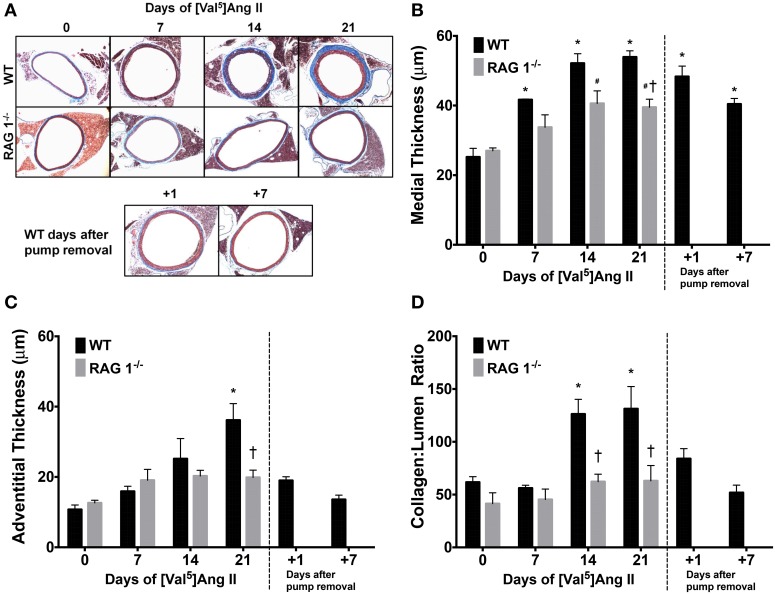
**Histomorphometrics of aortic cross-sections**. Representative trichrome images show aortic remodeling over the time course of Ang II treatment in WT and RAG 1^−/−^ mice and after pump removal in WT mice **(A)**. Medial **(B)** and adventitial **(C)** thicknesses were calculated using the trichrome stain. The ratio of collagen cross-sectional area to lumen area was measured by Picrosirius red staining **(D)**. ^*^P < 0.05 vs. day 0 of WT; ^#^*P* < 0.05 vs. day 0 of RAG 1^−/−^; ^†^*P* < 0.05 between different strains at each time point. *N* = 6. Data are means ± SEM.

Histological evaluation of aortas was performed in WT or RAG 1^−/−^ mice and representative sections are shown in Figure 4A. WT mice exhibited a significant increase in medial thickness by 14 days (Figure 4B). This increase, however, was significantly less prominent in RAG 1^−/−^ mice in comparison with WT mice (Figure 4B). The adventitial thickness increased significantly by day 21 in the WT mice but did not change in the RAG 1^−/−^ mice in comparison with the controls (Figure 4C). Collagen levels, represented by the collagen:lumen ratio as quantified by PSR staining, increased in the WT mice by day 14 but were not significantly increased in the RAG 1^−/−^ mice (Figure 4D). Additionally, 7 days after pump removal at 21 days of Ang II treatment, adventitial thickness and collagen content returned to day 0 levels. Only medial thickness remained significantly higher than control levels. These results indicate that aortic remodeling is significantly reduced in RAG 1^−/−^ mice in response to chronic Ang II infusion. These results further indicate a reversal of histological remodeling in the aorta after removal of the Ang II stimulus.

### Adoptive transfer of CD4^+^ T lymphocytes restores PWV in RAG 1^−/−^ mice

**Figure 5:**
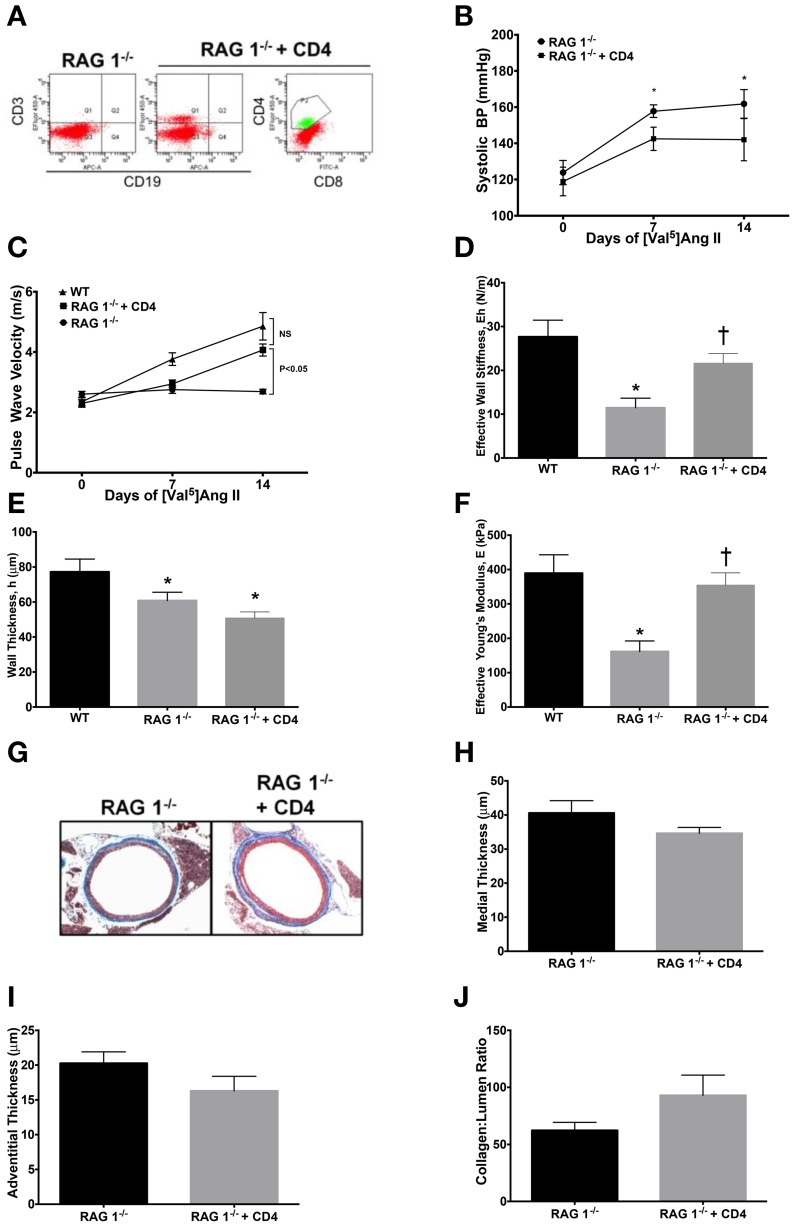
**Adoptive transfer of CD4^+^ T cells to RAG 1^−/−^ mice**. CD4^+^ T cells were isolated from CD8^−/−^ mice and adoptively transferred to RAG 1^−/−^ mice. **(A)** Representative splenic flow cytometry 2 weeks after adoptive transfer of CD4^+^ T cells to RAG 1^−/−^ mice is displayed. Systolic blood pressure measurements by tail cuff **(B)** were made in RAG 1^−/−^ with adoptively transferred CD4^+^ T cells and stiffness was measured by pulse wave velocity **(C)**. Parameters in **(D–F)** are at 14 days of Ang II, including **(D)** effective wall stiffness, **(E)** wall thickness, and **(F)** effective Young's modulus. Representative trichrome images **(G)** are presented of aortic cross-sections from RAG 1^−/−^ mice with adoptive transfer of CD4^+^ T cells followed by **(H)** medial and **(I)** adventitial thicknesses and **(J)** collagen:lumen ratio. ^*^P < 0.05 vs. day 0 of RAG 1^−/−^ in **(B)**; ^*^P < 0.05 vs. WT in **(D–F)**; ^†^*P* < 0.05 vs. RAG 1^−/−^. *N* = 6–8. Data are means ± SEM.

CD4^+^ T cells have been shown to be important in atherosclerosis (Dinh et al., 2012) and Ang II-induced thrombus formation (Senchenkova et al., 2011). Since RAG 1^−/−^ mice lack an adaptive immune system, including CD4^+^ T helper cells, we investigated whether adoptive transfer of CD4^+^ lymphocytes could restore vascular stiffness in response to Ang II. CD4^+^ cells were isolated from CD8^−/−^ mice and purified by IgG panning and 2 × 10^7^ cells were injected i.p. into RAG 1^−/−^ mice. Reconstitution was allowed to occur for 2 weeks; followed by infusion of Ang II. In order to account for diminishing numbers of transferred cells, mice were sacrificed after 2 weeks of Ang II infusion. Representative flow cytometry confirms adoptive transfer of CD4^+^ T cells that was devoid of cytotoxic T cells, characterized by CD8, or B cells, characterized by CD19 (Figure 5A).

CD4^+^ T cells did not significantly alter the arterial blood pressure in RAG 1^−/−^mice (Figure 5B). However, PWV increased significantly by 14 days in the RAG 1^−/−^mice receiving CD4^+^ lymphocytes (Figure 5C). The calculated wall stiffness increased in the RAG 1^−/−^ mice receiving CD4^+^ cells (Figure 5D), but wall thickness was not significantly affected (Figure 5E). Most importantly the effective Young's modulus also increased with CD4^+^ adoptive transfer in mice receiving Ang II (Figure 5F). Histological evaluation of aortic samples at 14 days of Ang II infusion showed no significant differences medial or adventitial thicknesses or collagen content in the vessels (Figures 5G–J). Therefore, adoptive transfer of T-lymphocytes reconstituted the ability of immune deficient mice to develop functional aortic vascular stiffness.

## Discussion

Vascular stiffness is an important predictor of cardiovascular disease events. Therefore, the elucidation of its mechanisms is imperative (Mitchell et al., 2010). Our study indicates that vascular stiffness is dependent on the collagen-mediated remodeling that is in-turn reliant on the adaptive immune system. Moreover, adoptive transfer of CD4^+^ T cells is able to restore stiffness in immunodeficient mice. We found in this study that histological parameters of aortic remodeling, namely increased collagen deposition as well as medial hypertrophy and adventitial fibrosis corresponded to an increase in PWV in immunocompetent WT mice over the period of chronic Ang II infusion. Furthermore, RAG 1^−/−^ mice displayed blunted histological remodeling and no increase in PWV despite a significant increase in the arterial blood pressure. These results are reinforced by a decrease in the diastolic fraction of the Doppler flow wave in the WT but not the RAG 1^−/−^ mice, supportive of a loss of Windkessel function in the immunocompetent mice. Moreover, the reflective wave propagation time decreased with Ang II administration in the WT but not the RAG 1^−/−^ mice, indicating increased wave reflection toward the heart in the WT mice. Increased wave reflection, in addition to arterial stiffening, is predictive of future cardiovascular disease events (Sung et al., 2012).

The results of our study support those of Wu et al. (2014), in which it was found that Ang II-induced aortic stiffening as measured by *ex vivo* biomechanical analysis was significantly increased in WT mice compared with immunodeficient RAG 1^−/−^mice. However, contrary to their results, we have found that blood pressure increased significantly in the RAG 1^−/−^ mice in response to [Val^5^]Ang II treatment. The PWV increased in the WT, did not change in the RAG 1^−/−^mice, and with reconstitution of the adaptive immune system the PWV increased to values similar to that of the WT mice receiving Ang II. Most notably, our functional stiffness measurements in RAG 1^−/−^ mice as measured by PWV include contributions of both active smooth muscle and structural alterations on vascular stiffening in a living system. This differs from biaxial biomechanical measurements, which only provide information on structural and material changes and are not translatable to the clinical setting. Our results showing that ablation of T lymphocytes, major constituents of adaptive immunity, prevents the development of aortic stiffening is consistent with a study showing that immunosuppression by Tregs reduces Ang II-induced stiffening in small arteries (Barhoumi et al., 2011) and large arteries (Majeed et al., 2014).

An interesting finding in our study is that pump removal after 21 days of Ang II treatment reduced PWV in the WT mice but the PWV stayed significantly elevated over pretreatment values. We suggest that this PWV level post-treatment is due to structural and material remodeling of the vessel wall. However, RAG 1^−/−^ mice did not exhibit raised PWV at all despite the effect of Ang II in raising blood pressure in this group significantly over the 3-week treatment. If the decrease in WT PWV after pump removal was due to the vasoconstrictive and blood pressure raising effects of Ang II, then that raises the question of why the RAG 1^−/−^ mice did not exhibit even a blunted increase in PWV over the treatment period. One possible explanation for this phenomenon is cellular repositioning of the smooth muscle that occurs in the WT mice (Martinez-Lemus et al., 2009) and altered interactions between the ECM and the smooth muscle (Schiffrin, 2012). Moreover, vascular stiffening and hypertension might not always be related (Weisbrod et al., 2013). These possibilities must be investigated in future studies.

In order to further explore to the question of whether heightened blood pressure and vascular stiffening are related, Wu et al. (2014) utilized hydralazine and hydrochlorothiazide to prevent the Ang II-mediated increase in blood pressure and found no resulting increase in vascular stiffness. Although, they concluded from these observations that blood pressure and mechanical stress alone could cause vascular stiffening, hydralazine has anti-neoplastic properties and prevents tissue growth since it is a non-nucleoside DNA methyltransferase (DNMT) inhibitor (Graca et al., 2014). Therefore, hydralazine's potential anti-proliferative effect as a DNMT inhibitor is a confounding factor for the interpretation of these results. Moreover, we have found in our study that the RAG 1^−/−^ mice do not exhibit an increase in aortic stiffness despite a significant elevation in blood pressure.

In our study, CD4^+^ T cells were able to restore the Ang II-induced increase in PWV in RAG 1^−/−^ mice but were not able to change collagen levels or aortic morphometry by histology. Our histological findings are consistent with Wu et al. (2014). However, this group did not find a restoration of stiffness with CD4^+^ T lymphocyte adoptive transfer to RAG 1^−/−^ mice. Differences may be due to differing methods used to purify and adoptively transfer CD4^+^ T cells and to measure aortic stiffness.

Mechanisms for this restoration of Ang II-induced arterial stiffening by CD4^+^ T cells could involve increases in crosslinks, such as advanced glycation end-products that are known to reduce arterial compliance (Kass et al., 2001). Enzyme-mediated crosslinks such as those by lysyl oxidases and transglutaminases that can lead to stiffening (Schiffrin, 2012; Eberson et al., 2015) may also be regulated by CD4^+^ T cells. Further mechanisms could include upregulation of inflammatory mediators such as adhesion molecules, cytokines, and intracellular transcription factors as well as mediators of oxidative stress. Adding on, remodeling of the extracellular matrix, whose fibrillar components create altered interactions with smooth muscle cells, could facilitate reduction of vascular distensibility (Schiffrin, 2012).

The contribution of the immune system to vascular stiffening is indeed a critical area of investigation that is important for the clinical setting. It has been shown that reduced carotid arterial distensibility among HIV-infected women, adjusted for age, HIV medications, and vascular risk factors, is associated with higher frequencies of CD4^+^CD38^+^HLA^−^DR^+^ T cells (Kaplan et al., 2011). This suggests that pro-inflammatory T cells may produce vascular remodeling in HIV-infected patients leading to arterial stiffening. In another clinical example, rheumatoid arthritis (RA) in young patients is associated with shortened pulse propagation time (Yildiz et al., 2004) and anti-tumor necrosis factor α therapy reduces aortic stiffening and inflammation in RA patients (Maki-Petäjä et al., 2012).

In summary, we have shown that vascular stiffness does not increase in response to Ang II treatment in RAG 1^−/−^ mice despite an increase in blood pressure. Furthermore, adoptive transfer of CD4^+^ T cells restores the Ang II-mediated increase in aortic stiffness. Our results use *in vivo* measures of vascular stiffening in mice and support the role of the adaptive immune system, specifically CD4^+^ T cells, in the development of vascular stiffness and show a disparity in the role of blood pressure regulation on vascular stiffness.

### Conflict of interest statement

The authors declare that the research was conducted in the absence of any commercial or financial relationships that could be construed as a potential conflict of interest.
